# Identification and Fine Mapping of *RppM*, a Southern Corn Rust Resistance Gene in Maize

**DOI:** 10.3389/fpls.2020.01057

**Published:** 2020-07-09

**Authors:** Shuai Wang, Ruyang Zhang, Zi Shi, Yanxin Zhao, Aiguo Su, Yuandong Wang, Jinfeng Xing, Jianrong Ge, Chunhui Li, Xiaqing Wang, Jidong Wang, Xuan Sun, Qian Liu, Yining Chen, Yunxia Zhang, Shuaishuai Wang, Wei Song, Jiuran Zhao

**Affiliations:** Maize Research Center, Beijing Academy of Agriculture and Forestry Sciences (BAAFS), Beijing Key Laboratory of Maize DNA Fingerprinting and Molecular Breeding, Beijing, China

**Keywords:** maize, southern corn rust, fine mapping, resistance gene, *Puccinia polysora*

## Abstract

Southern corn rust (SCR) caused by *Puccinia polysora* Underw. is a major disease causing severe yield losses during maize production. Here, we identified and mapped the SCR resistance gene *RppM* from the near-isogenic line Kangxiujing2416 (Jing2416K), which harbors *RppM* in the genetic background of the susceptible inbred line Jing2416. In this study, the inheritance of SCR resistance was investigated in F_2_ and F_3_ populations derived from a cross between Jing2416K and Jing2416. The observed 3:1 segregation ratio of resistant to susceptible plants indicated that the SCR resistance is controlled by a single dominant gene. Using an F_2_ population, we performed bulked segregant analysis (BSA) sequencing and mapped *RppM* to a 3.69-Mb region on chromosome arm 10S. To further narrow down the region harboring *RppM*, we developed 13 insertion/deletion (InDel) markers based on the sequencing data. Finally, *RppM* was mapped to a region spanning 110-kb using susceptible individuals from a large F_2_ population. Two genes (*Zm00001d023265* and *Zm00001d023267*) encoding putative CC-NBS-LRR (coiled-coiled, nucleotide-binding site, and leucine-rich repeat) proteins, a common characteristic of R genes, were located in this region (B73 RefGen_v4 reference genome). Sequencing and comparison of the two genes cloned from Jing2416K and Jing2416 revealed sequence variations in their coding regions. The relative expression levels of these two genes in Jing2416K were found to be significantly higher than those in Jing2416. *Zm00001d023265* and *Zm00001d023267* are thus potential *RppM* candidates.

## Introduction

Southern corn rust (SCR), caused by the fungus *Puccinia polysora* Underw., can lead to poor yields and decreased nutritional quality in maize (*Zea mays* L.). A disastrous plant disease, SCR is widely established in warm-temperate and tropical regions, such as the United States, Asia, and Africa (Liu et al., 2003; Dolezal et al., 2009; Brewbaker et al., 2011). SCR has been prevalent in western Africa since 1949, where it has been responsible for yield losses as high as 50% (Rhind et al., 1952; Stanton and Cammack, 1953). In the United States, SCR-induced yield losses up to 17.7% and 39.1% have been reported in inoculated field sites in Pennsylvania and Maryland, respectively (Raid et al., 1988). In recent years, SCR has become the major disease in maize-producing regions of the world, resulting in significant crop losses (Ali and Yan, 2012). In China, SCR was first identified in Sanya and Ledong, Hainan Province, in the early 1970s (Duan and He, 1984) and has gradually spread to high-latitude areas because of global climate change (Wang et al., 2019). In 1998, an outbreak and epidemic of SCR in northern China resulted in yield losses of 42% to 53% (Zhou et al., 2008). The lack of resistance in temperate germplasm is the main reason for SCR epidemics (Rodriguez-Ardon et al., 1980; Bailey, 1987; Brewbaker et al., 2011). The identification and utilization of new SCR resistance sources is therefore essential in current maize breeding programs.

Genetic studies on SCR resistance began as early as the 1950s. At least 10 races of *P. polysora* (EA.1, EA.2, EA.3, and PP.3-PP.9), which can be distinguished by the reactions they induce on different maize lines, have been documented (Storey and Ryland, 1954; Ryland and Storey, 1955; Ullstrup, 1965). Several unique, major, race-specific SCR-resistance genes were identified in maize in earlier studies, including *Rpp1* (conferring resistance to *P. polysora* races EA.1 and EA.3), *Rpp2* (conferring resistance to races EA.1, EA.2, and EA.3), and *Rpp9* (conferring resistance to race PP.9) (Storey and Howland, 1959; Storey and Howland, 1967). *Rpp1* (a fully dominant gene) and *Rpp2* (an partial dominant gene) were identified from Mexican line AFER029 and Colombian line AFR024, respectively (Storey and Howland, 1957). The two genes were found to be loosely linked to each other, but their genomic locations had not been confirmed (Storey and Howland, 1959). The single dominant gene *Rpp9* from the African line PI186208 is closely linked, with a genetic distance of 1.5 cM, to the common rust (*Puccinia sorghi* *Schw*.) resistance gene *Rp1* on chromosome 10S (Ullstrup, 1965; Brewbaker et al., 2011). Although *Rpp9* has been successfully used to control SCR for 30 years, the resistance of *Rpp9*-harboring cultivars has been lost because of the high genetic variability of SCR races in the southern United States (Dolezal et al., 2009). Major effect genes for resistance to SCR in different maize germplasm resources have been reported (Futrell et al., 1975; Scott et al., 1984; Holland et al., 1998). These genes are also located on chromosome 10S and closely linked to *Rpp9*, but their allelic relationship with *Rpp9* has not been determined (Casela and Ferreira, 2002).

With the availability of genome sequencing and abundant genetic markers, a number of SCR-resistance genes have been mapped. Quantitative trait loci (QTLs) for SCR resistance have been detected on maize chromosomes 3, 4, 8, 9 and 10 from different resistant germplasm resources, but discrepancies exist among different study findings (Holland et al., 1998; Jiang et al., 1999; Brunelli et al., 2002; Wisser et al., 2006; Jines et al., 2007). Liu et al. (2003) confirmed that the inheritance of SCR resistance in maize inbred line P25 is controlled by a major resistance gene, which was mapped to chromosome 10S at a distance of 5.8 cM from the simple sequence repeat (SSR) marker phi059. In a subsequent study, *RppP25* was fine mapped to a 40-kb region between SSR marker P091 and insertion/deletion (InDel) marker M271, and *GRMZM2G060884* encoding a putative nucleotide-binding site leucine-rich repeat (NBS-LRR) protein was identified as the candidate gene (Zhao et al., 2013). Another single dominant SCR-resistance gene, *RppQ*, has been identified from inbred line Qi319; this gene has also been mapped on chromosome 10S between the sequence-characterized amplified region MA7 and the amplified fragment length polymorphism marker M-CCG/E-AGA_157_, with genetic distances of 0.46 and 1.71 cM, respectively (Chen et al., 2002; Chen et al., 2004; Zhou et al., 2008). In addition, Zhang et al. (2010) indicated that the inheritance of SCR resistance in inbred line W2D is due to a single dominant gene, *RppD*, which was mapped on chromosome 10S between SSR marker umc1291 and the cleaved-amplified polymorphic sequence marker CAPS858, with distances of 2.9 and 0.8 cM, respectively. According to an allelism test, the *RppD* locus is different from *RppQ* and *RppP25*. Wu et al. (2015) identified a single dominant gene, *RppS*, from the tropical inbred line SCML205 that conferred broad resistance against SCR. An allelism test indicated that *RppS* was not allelic to *RppQ*, *RppD* or *RppP25* but was possibly linked with them.

The objectives of the present study were to (1) evaluate the inheritance of SCR resistance in F_2_ and F_3_ populations derived from reciprocal crosses between resistant near-isogenic line Jing2416K and susceptible line Jing2416, (2) fine map the SCR-resistance gene *RppM* using combined bulked segregant analysis (BSA) sequencing and traditional linkage analysis, and (3) predict the candidate gene of *RppM* using sequencing and expression data. The above studies establish a foundation for future isolation and cloning of the *RppM* candidate gene.

## Materials and Methods

### Plant Materials

The elite inbred line 1484, which exhibits complete resistance to SCR, was used as the donor parental line in this study. The SCR-susceptible line Jing2416, which has been extensively used in maize production in China, was the recurrent parent. After an initial cross between 1484 and Jing2416, six rounds of backcrossing and phenotypic selection were performed to generate BC_6_ families. Evaluation of resistance and background selection were conducted on each BC_6_ family to identify a single resistant individual with the highest proportion of the recurrent parental genome. The selected BC_6_ individuals were self-pollinated to obtain homozygous resistance loci. Finally, a near-isogenic line of Jing2416 carrying the resistance gene allele from 1484 was developed (**Supplementary Figure S1**) and designated as KangxiuJing2416 (Jing2416K). The F_2_ population, derived from reciprocal cross between Jing2416K and Jing2416, was used for genetic analysis and fine mapping of *RppM*.

### Evaluation of SCR Resistance

All plants were grown at the experimental station of the Beijing Academy of Agriculture and Forestry Sciences in Sanya, Hainan Province, China. In Sanya, SCR develops naturally and becomes more severe as maize plants mature. One row of the susceptible parent Jing2416 was grown as the susceptible control per 20 rows. The parents and F_1_, F_2_, and F_3_ populations were identified by natural inoculum, and SCR resistance ratings were recorded at the grain filling stage. Using the parental lines Jing2416 and Jing2416K as controls, plants were classified as resistant or susceptible according to a five-point rating scale described by Zhao et al. (2013).

### Genetic Analysis

Each individual in the F_2_ population was classified as resistant or susceptible based on its response to the SCR pathogen, with the parental lines used as controls. χ^2^ goodness-of-fit tests were applied to determine whether segregation in the F_2_ populations corresponded to a 3:1 ratio. If the *P*-value was greater than the level of significance (0.05), the segregation ratio was considered to fit the expected ratio in the populations.

### DNA Extraction, Library Construction and Whole-Genome Re-Sequencing

Genomic DNA was extracted from leaves of parental plants and F_2_ individuals using the cetyltrimethylammonium bromide method (Murray and Thompson, 1980) with minor modifications. The isolated DNAs of 45 resistant and 45 susceptible plants from the F_2_ population and the two parents were quantified by 1% agarose gel electrophoresis with ethidium bromide staining and then more precisely quantified on a Qubit 2.0 fluorometer (Life Technologies, CA, USA).

DNA of resistant and susceptible individuals was taken from single plant and then equal amounts were respectively mixed to prepare resistant and susceptible pools (Liu et al., 2019). We abbreviate Jing2416 as R01, Jing2416K as R02, resistant pool as R03 and susceptible pool as R04 for simplified description. DNAs of four samples (R01, R02, R03, and R04) were randomly broken into 350-bp fragments by ultrasonication. After end repair and addition of nucleotide (A) overhangs, the fragments were ligated with sequencing adapters using T4 DNA ligase and then amplified by PCR. The purified products were used to construct a sequencing library, which was loaded onto an Illumina sequencing platform (Illumina, San Diego, CA, USA) for paired-end sequencing following the manufacturer’s recommendations.

### BSA Combining SNP-Index and InDel-Index Association Analysis

The raw sequencing data were processed to obtain clean reads as described previously (Liu et al., 2019). Clean reads were aligned to the maize B73 RefGen_v4 reference genome (https://www.maizegdb.org/gbrowse) using the Burrows-Wheeler Aligner (Li and Durbin, 2009), and Picard (https://sourceforge.net/projects/picard/) was used to mark duplicates. Highly accurate SNP and InDel sets were obtained using GATK (Mckenna et al., 2010) and a range of filters (Reumers et al., 2012). An association analysis was performed using both SNP-index (Abe et al., 2012) and InDel-index (Singh et al., 2017) methods.

### Fine Mapping of *RppM*

To genotype the parents and 710 susceptible F_2_ individuals, 13 InDel markers (**Supplementary Table S1**) were designed in Primer 5.0 using the parental re-sequencing data. Genotypes were characterized by capillary zone electrophoresis. Each 20-μl PCR mixture contained approximately 100 ng of template DNA, 0.25 μM each of forward and reverse primers, 2× *Taq* Plus Master Mix (Vazyme Biotech, Beijing, China) and ddH_2_O. The PCR amplification protocol was as follows: denaturation at 95°C for 5 min, followed by 35 cycles of denaturation at 95°C for 40 s, annealing at 57°C for 35 s and extension at 72°C for 45 s, with a final extension step at 72°C for 10 min.

### Coding Sequence (CDS) Sequencing and Alignment

Target-region sequences were analyzed using the Maize Genetics and Genomics database (https://www.maizegdb.org/gbrowse) and the FGENESH online tool (http://linux1.softberry.com/berry.phtml). Markers specific for CDSs of ORFs in the candidate region were designed using maize B73 reference sequences and the re-sequencing data (**Supplementary Table S2**). After PCR amplification, the products were purified using a Zymoclean Gel DNA Recovery kit (Zymo, Beijing, China) and cloned into a T-vector with a pEASY-Blunt Simple Cloning kit (Trans). After PCR detection, six positive clones were picked out for Sanger sequencing, respectively. Sequence alignments were conducted using DNAMAN v6.0 (http://www.lynnon.com/), and major functional domains were predicted using the Simple Modular Architecture Research Tools (SMART) program (http://smart.embl-heidelberg.de/).

### Quantitative Real-Time PCR Analysis

For expression analysis of *Zm00001d023265* and *Zm00001d023267*, total RNA was extracted using an RNAprep Pure Plant kit (Tiangen) from ear leaves of Jing2416K and Jing2416 at three time points: 48 Days After Sowing (DAS) (before the appearance of visible lesions in ear leaves of Jing2416), 55 DAS (lesions just visible) and 66 DAS (lesions clearly visible). First-strand cDNA was synthesized using a SuperScript II kit (Takara) following the manufacturer’s recommendations. Primer pairs were designed using GenScript (https://www.genscript.com/tools/real-time-pcr-taqman-primer-design-tool) and are listed in **Supplementary Table S3**. Quantitative real-time PCR amplifications were carried out using a SYBR Premix Ex *Taq* kit (Takara) as described previously (Wang et al., 2017), with the maize *Actin* gene (*Zm00001d012277*) used as an internal reference. Relative expression levels of the two genes were determined by the 2^−ΔΔCT^ method (Livak and Schmittgen, 2001).

## Results

### Characterization of the Resistant Near-Isogenic Line

**Figure 1:**
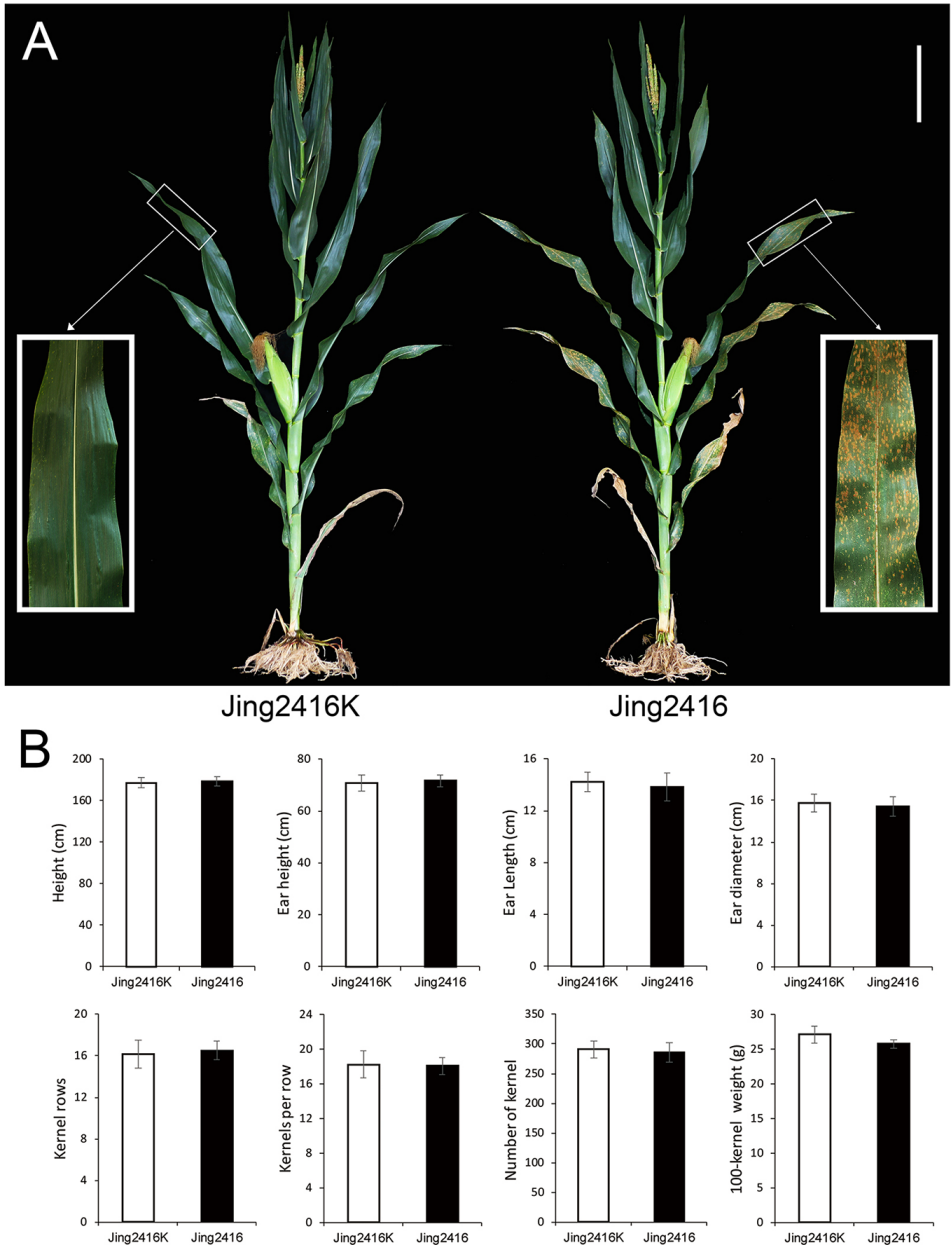
Comparison of Jing2416K and Jing2416 plants. **(A)** Evaluation of resistance to SCR in Jing2416K and Jing2416 at the filling stage. Scale bar: 25cm. **(B)** Statistical analysis of agronomic traits of Jing2416K and Jing2416 plants. Data are means ± SD of 12 plants.

Evaluation of SCR resistance was performed by natural inoculum in Hainan Province. Relatively small reddish-brown lesions started to appear on the lower leaf surfaces of Jing2416 at the tasseling stage; disease symptoms then spread rapidly to leaves on the rest of the plant. The lesions increased in both number and size at the filling stage, with subsequent leaf chlorosis and rapid senescence of the whole plant at the late maturity stage (**Figure 1A**). In contrast, Jing2416K plants were immune, with no lesions appearing (**Figure 1A**). Other than resistance to SCR, agronomic traits of susceptible line Jing2416 were basically the same as those of resistant line Jing2416K (**Figure 1B**).

### Genetic Analysis of SCR Resistance

Reciprocal crosses were made between resistant line Jing2416K and susceptible line Jing2416, and the F_1_ plants exhibited the resistant phenotype. Among the 205 F_2_ individuals from the cross of Jing2416 × Jing2416K, 153 showed disease resistance, while the remaining 52 were susceptible; there are 187 resistance plants and 59 susceptible plants among the 246 F_2_ individuals from the cross of Jing2416K × Jing2416 (**Table 1**). The segregation of the F_2_ populations fit a 3:1 (χ^2^ = 0.0016/0.0866 < 3.84, *p* = 0.904/0.713) resistant to susceptible phenotypic ratio. This result indicates that the inheritance of SCR resistance in inbred line Jing2416K is controlled by a single dominant gene, which we designated as *RppM*.

**Table 1:** Genetic analysis of resistance to southern corn rust.

F_2_ population	Total	Resistant	Susceptible	Segregation ratio	χ^2^	*P*-value
Jing2416 × Jing2416K	205	153	52	3.19	0.0016	0.904
Jing2416K × Jing2416	246	187	59	3.07	0.0866	0.713

### Preliminary Mapping of *RppM*

To determine the location of *RppM*, we used a large F_2_ population obtained from the cross of Jing2416 × Jing2416K. Two DNA pools, a resistant pool (45 F_2_ individuals with the resistant phenotype, 30 homozygous resistant and 15 heterozygous F_2_ genotypes) and a susceptible pool (45 F_2_ individuals with the susceptible phenotype), were constructed for use in BSA sequencing. A total of 276.63 Gb of clean data of high quality (93.12% < Q30 < 93.54%) and stable GC content (45.73% < GC < 46.06%) were obtained by Illumina sequencing; the average sequencing depth was 23.5× for the two parents and 32.5× for the two DNA pools (**Supplementary Table S4**). These high-quality data served as a robust foundation for subsequent analysis.

**Figure 2:**
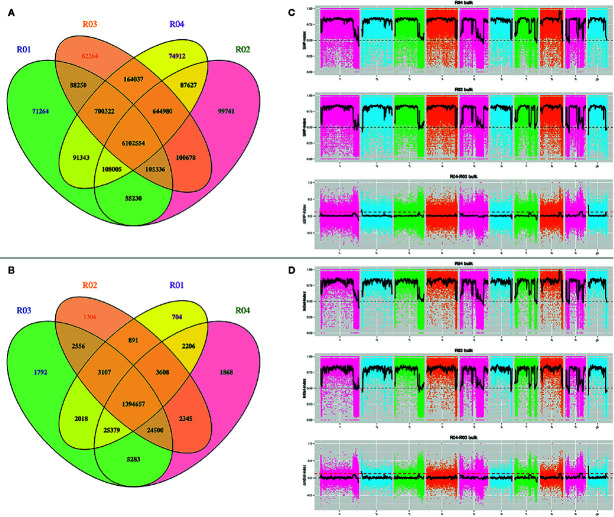
Preliminary mapping of *RppM* by BSA sequencing. **(A)** Venn diagram of SNP in the four pools. **(B)** Venn diagram of InDel in the four pools. **(C)** SNP-index algorithm to map *RppM*. **(D)** InDel-index algorithm to map *RppM*. R01: Jing2416; R02: Jing2416K; R03: the resistant pools; R04: the susceptible pools.

**Figure 3:**
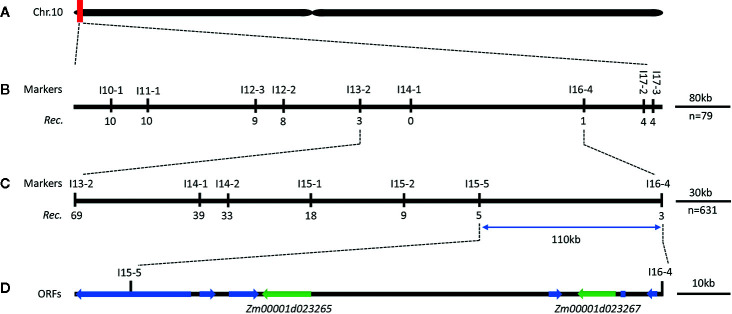
Genetic and physical maps of the *RppM* gene. **(A)** The *RppM* gene was located to a region of 3.69 Mb on short arm of chromosome 10. **(B)** The *RppM* gene was delimited to the I13-2 and I16-4 interval using 79 F_2_ susceptible individuals; marker names and number of recombinants are shown. The number below each marker is the number of recombinants between that marker and *RppM*. **(C)** Fine genetic mapping of the *RppM* gene based on 631 susceptible F_2_ individuals. **(D)** Eight putative ORFs were located in an ~110-kb region.

The above reads were mapped onto the maize B73 RefGen_v4 reference genome. The average mapping rate was 90.1% for the four samples. A total of 1,877,747 and 841,755 SNPs, of which 24,120 and 4,367 were respectively non-synonymous, were obtained from the parents and the two mixed pools, respectively. Notably, we detected 325,308 and 109,918 small InDels in total among the parents and the two mixed pools. As shown by the Venn diagrams in **Figures 2A, B**, the four samples shared the same alleles at 6,102,554 SNP and 1,394,657 InDel loci. Resistance loci were then identified by combining SNP-index and InDel-index association algorithms. Taking overlapping regions into account, loci were mapped onto eight regions on chromosomes 2, 9, and 10 (**Figures 2C, D**; **Supplementary Table S5**). Polymorphic InDel markers developed in the 0-3,690,000-bp (3.69 Mb) region of chromosome 10S showed linkage to *RppM* (**Figures 3A, B**; **Supplementary Table S1**). A total of 754 genes, including 70 non-synonymous and 10 frameshifted ones, were annotated in the associated region.

### Fine Mapping of *RppM*

For the fine mapping of the *RppM* locus, eight InDel polymorphic markers developed in the candidate region were used for a set of 79 susceptible F_2_ individuals, we localized the resistance locus between markers I13-2 and I16-4 (**Figure 3B**; **Supplementary Table S1**). Further genotyping of an additional 631 susceptible F_2_ individuals derived from the same cross with five additional polymorphic markers, we finally anchor *RppM* to a 110-kb region between InDel markers I15-5 and I16-4 (**Figure 3C**; **Supplementary Table S1**). All of recombinants were self-pollinated to obtain F_2:3_ plants, which also exhibited the susceptible phenotype. Based on the maize B73 RefGen_v4 reference (https://www.maizegdb.org/gbrowse), the 110-kb region in Jing2416K and Jing2416 contained eight predicted open reading frames (ORFs) (**Figure 3D**; **Table 2**).

**Table 2:** The eight open reading frames in the target 110-kb region.

Gene	Gene ID	Physical locus	Description
ORF1	*Zm00001d023262*	1565392-1599381	protein ROOT PRIMORDIUM DEFECTIVE 1-like; brick3
ORF2	*Zm00001d023263*	1592479-1594283	glutamine-fructose-6-phosphate transaminase (isomerizing)
ORF3	*Zm00001d023264*	1600459-1605134	Aladin
ORF4	*Zm00001d023265*	1606001-1612705	putative CC-NBS-LRR protein
ORF5	*Zm00001d023266*	1670707-1671857	aladin
ORF6	*Zm00001d023267*	1680751-1687113	putative CC-NBS-LRR protein
ORF7	*Zm00001d023268*	1689570-1689643	tRNA
ORF8	*Zm00001d023269*	1698336-1698995	protein contain the VQ motif

### Evaluation of *RppM* As a Candidate Gene

**Figure 4:**
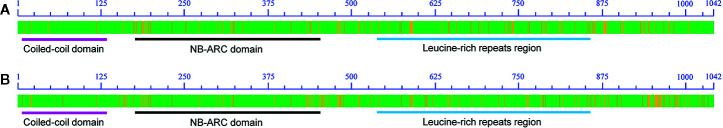
Alignment of the conserved motifs of the *RppM* candidate genes. **(A, B)** Diagrams of amino acid sequence alignments of Zm00001d023265 **(A)** and Zm00001d023267 **(B)**. Matching sequence regions are indicated in green, and variable regions are shown in yellow. Coiled-coil, NB-ARC and leucine-rich-repeat domain regions are underlined with violet, black and blue, respectively.

Among the eight predicted ORFs, two genes *Zm00001d023265* and *Zm00001d023267* were annotated as genes encoding putative CC-NBS-LRR proteins that had a domain architecture that included an N-terminal coiled-coil domain, a nucleotide-binding domain, and leucine-rich repeats—a common R gene characteristic. *Zm00001d023262* (*brick3*), the only one of the eight ORFs that has been previously cloned, promotes polarized cell division and cell morphogenesis in the maize leaf epidermis and has no connection with resistance (Facette et al., 2015). The CDSs of the remaining genes in the 110-kb region were cloned and sequenced. The only variation observed between the two parents was in the CDSs of *Zm00001d023265* and *Zm00001d023267*. A variance of 4.95% (Number of variant base pair: 155; Total number of base pair: 3131) was observed in the CDS of *Zm00001d023265* between Jing2416 and Jing2416K, and the amino acid sequence differed by 9.68% (Number of variant amino acid: 101; Total number of amino acid: 1042) (**Figure 4A**; **Supplementary Figure S2**). In regard to *Zm00001d023267*, the CDS and amino acid sequence varied by 5.52% (Number of variant base pair: 173; Total number of base pair: 3134) and 11.02% (Number of variant amino acid: 115; Total number of amino acid: 1043), respectively, between the two maize lines (**Figure 4B**; **Supplementary Figure S3**). The CDSs of both *Zm00001d023265* and *Zm00001d023267* were found to consist of 3,129 nucleotides and encode a putative 1,042-amino-acid protein (**Figure 4**).

**Figure 5:**
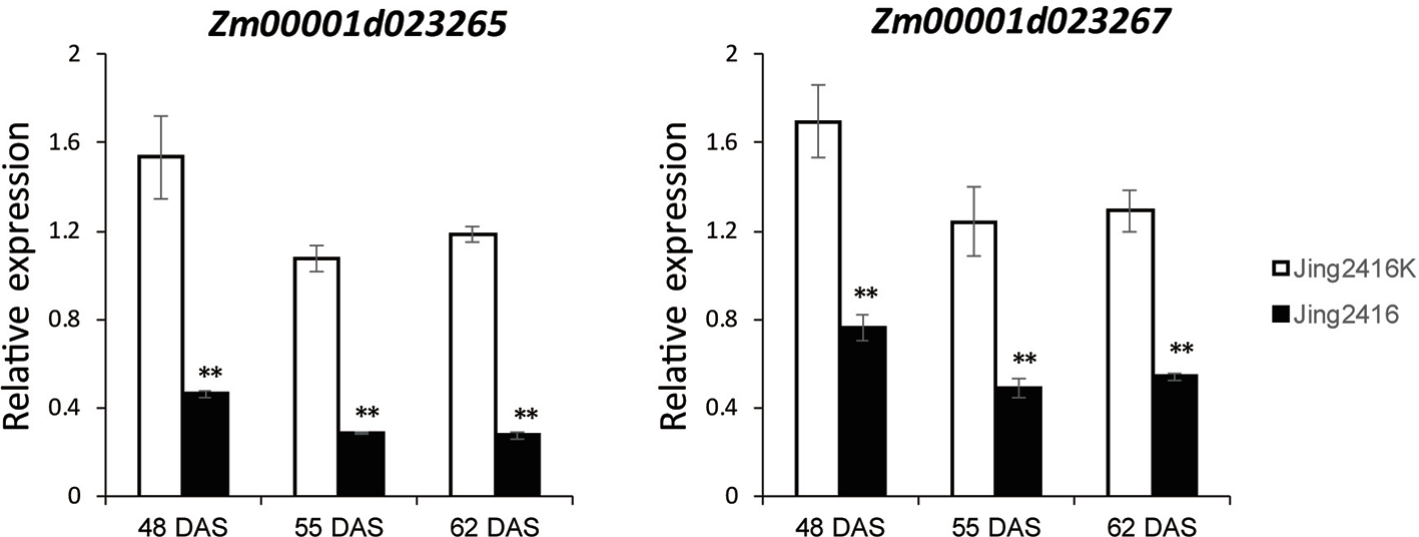
Expression analysis of *Zm00001d023265* and *Zm00001d023267*. RNA was extracted from ear leaves of Jing2416K and Jing2416 at 48, 55 and 66 DAS, respectively. Data represent means ± SD of three biological replicates. (Student’s *t*-test: **, *P* < 0.01).

To determine the relationship of *Zm00001d023265* and *Zm00001d023267* to SCR resistance, we examined their expressions in Jing2416K and Jing2416 ear leaves by quantitative real-time PCR (qRT-PCR) at three time points: 48 DAS (before the appearance of visible lesions in ear leaves of Jing2416), 55 DAS (lesions just visible) and 66 DAS (lesions clearly visible). At 48 DAS, expression levels of *Zm00001d023265* and *Zm00001d023267* in Jing2416K ear leaves were 3.30- and 2.22-fold, respectively, of those in Jing2416; at 55 DAS, expressions of the two genes in Jing2416K were increased by 3.74- and 2.53-fold, respectively, compared to Jing2416; and at 62 DAS their expressions were still higher than those of Jing2416 by 4.30- and 2.37-fold, respectively (**Figure 5**). Taken together, these results point to *Zm00001d023265* and *Zm00001d023267* as potential *RppM* candidates.

## Discussion

With more than 7,000 species, rust fungi constitute the largest group of plant fungal pathogens and are difficult to control and manage because of their rapid evolution, dynamic population structure, and wide dispersal of anemochorous spores (Kangasjarvi et al., 2012; Rochi et al., 2016; Gill et al., 2018). Several of these pathogens are responsible for three rust diseases in maize: SCR, common rust, and tropical rust. *Puccinia polysora*, a biotrophic fungal pathogen, causes SCR (McGee, 1988). SCR has become one of the most destructive plant diseases in the tropics and subtropics. In China, SCR has gradually spread to high-latitude regions following global warming, where it causes significant yield losses in maize production areas every year (Wang et al., 2019). A few inbred lines from tropical germplasm are resistant to SCR, whereas almost all temperate germplasm resources are susceptible (Zhao et al., 2013). Therefore, the incorporation of elite tropical inbred lines as resistant sources would provide underexploited germplasm for maize breeding and germplasm improvement in temperate regions (Nelson and Goodman, 2008) and thus enhance the SCR resistance of maize hybrids.

The races of *Puccinia polysora* have not yet been classified in China. In the current study, we evaluated SCR resistance under natural field conditions using resistant parent Jing2416K and susceptible parent Jing2416 as controls. Previous studies have shown that this approach is effective and economical (Zhou et al., 2008; Zhang et al., 2010). For example, Holland et al. (1998) confirmed that environmental effects on phenotypic variation can be controlled though replication and progeny testing by evaluating SCR resistance in multiple environments depending on natural inoculum. Jines et al. (2007) scored 143 top crosses for SCR in four environments relying upon natural inoculum and suggested that the inheritance of SCR resistance was simple in nature. In the present study, we carried out phenotypic characterization of SCR resistance in different field trials in Sanya over multiple years. The typical conditions at Sanya—high relative humidity and warm temperatures—are favorable for the development and spread of SCR (Liu et al., 2003; Zhang et al., 2010; Zhao et al., 2013). Jing2416K was immune to SCR, and F_1_ plants from reciprocal crosses between Jing2416K and Jing2416 were highly resistant in all trials; in contrast, Jing2416 plants exhibited significant susceptibility (**Figure 1**). The segregation ratio of resistant to susceptible individuals in both F_2_ populations fit the expected ratio of 3:1 in all trials. In addition, phenotypic evaluation of F_3_ individuals was used to precisely determine the genotypes of recombinants at the *RppM* locus.

Decades of research have shown that traditional map-based cloning is an efficient method for isolating genes/QTLs responsible for target traits (Zuo et al., 2015; Lu et al., 2017; Zhang et al., 2017a). Usually, a molecular genetic map of a designated population (F_2_, double haploid or recombinant inbred line population) based on hundreds of genetic markers is constructed for primary mapping; new markers in the primary mapping region are then developed to narrow down the region according to the genotype of recombinants to a sufficient size to screen for candidate genes. This process therefore always requires a considerable amount of time (Zhu et al., 2013; Fan et al., 2016). Although mapping by next-generation sequencing can generally only detect the target interval at the Mb level (Haase et al., 2015; Zheng et al., 2016; Kayam et al., 2017) because of insufficient meiotic recombination events in the mixed pool which typically contains approximately 30 to 100 individuals, such an approach remains an efficient and reliable method for primary mapping compared with genetic map construction (Schneeberger, 2014). In the present study, the SCR-resistance gene *RppM* was primarily mapped to a 3.69-Mb region by BSA sequencing combined with SNP-index and InDel-index analyses (**Figure 2**). The target region of *RppM* was then narrowed down to a 110-kb interval depending on the new markers developed within the primarily mapping region by traditional linkage analysis (**Figure 3**). Such a method enables faster, more accurate fine mapping of genes/QTLs using a large F_2_ population, especially when traits are controlled by single nuclear-encoded genes/QTLs. Finally, we performed progeny testing and replication to ensure accurate determination of phenotypes.

Previous genetic studies have identified several SCR-resistance genes using different maize germplasm sets. Most of these genes, including *RppD*, *RppS*, *RppQ*, and *Rppp25*, reside in clusters on the short arm of chromosome 10 (Ullstrup, 1965; Scott et al., 1984; Holland et al., 1998; Chen et al., 2004; Jines et al., 2007; Zhang et al., 2010). The common rust resistance genes *Rp1* and *Rp5* have been located in this region as well (Hulbert and Bennetzen, 1991). These SCR-resistance genes have been roughly mapped to chromosome 10S, and none have been cloned. Zhang et al. (2010) reported that the *RppD* locus was different from but tightly linked to *RppQ* and *RppP25* according to an allelism test, while the allelic relationships of other resistance genes were not determined. *RppM* is probably closely linked or even allelic to other SCR-resistance genes identified on chromosome 10S. Among them, *Rppp25* is the only SCR-resistance gene that has been fine mapped to a 40-kb region based on the B73 RefGen_v2 reference genome corresponding to a 96.5-kb region (Chr10:2,651,981–2,748,521) on the B73 RefGen_v4 reference genome (Zhao et al., 2013). In our study, *RppM* was ultimately anchored to a 110-kb region (Chr:1,586,659–1,697,392) on the B73 RefGen_v4 reference genome with a physical distance 1 Mb away from *Rppp25*.

To date, numerous R genes against various pathogens have been cloned and characterized from a variety of plant species, the most predominant of which encode proteins generally containing a putative amino-terminal signaling domain, a nucleotide-binding site (NBS) and a series of carboxy-terminal leucine-rich repeats (LRRs) (Meyers et al., 2005; Ma et al., 2015; Zhang et al., 2017b). The NBS domain is probably involved in nucleotide binding, hydrolyzation, and the induction of immunity responses, whereas the LRR domain is potentially responsible for protein–protein interactions (Martin et al., 2003; Collier and Moffett, 2009; Chen et al., 2011). These NBS-LRR proteins have been divided into two major types on the basis of their N-terminal domains: TIR-NBS-LRR or TNL proteins that contain an amino-terminal Toll/interleukin receptor domain, and CC-NBS-LRR or CNL proteins that contain an amino-terminal coiled-coiled motif (Soriano et al., 2005; Qi et al., 2012). Moreover, R genes are well known to form gene clusters on chromosomes, usually as the result of tandem duplications of paralogous sequences (Meyers et al., 2005; Strange and Scott, 2005; Wang et al., 2007). In our study, eight ORFs were identified in the 110-kb candidate region based on the B73 RefGen_v4 reference genome; two of them, *Zm00001d023265* and *Zm00001d023267*, encode putative typical CC-NBS-LRR proteins. Molecular cloning and sequence analysis confirmed variation was present between the two parents in the CDS and protein sequence of the two genes (**Figure 4**). The results of qRT-PCR analysis indicated that the expressions of *Zm00001d023265* and *Zm00001d023267* in resistant line Jing2416K were significantly higher than those in susceptible line Jing2416 at 48, 55 and 66 DAS (**Figure 5**). We thus conclude that *Zm00001d023265* and *Zm00001d023267* are the most likely *RppM* gene candidates. The location and candidate gene screening of *RppM* could lay a robust foundation for later cloning the *RppM* gene and its application in marker-assisted selection for the breeding of maize varieties with high SCR resistance.

## Data Availability Statement

The sequencing data has been uploaded to Sequence Read Archive (SRA) (Submission ID: SUB7638157, SRA accession: PRJNA641879).

## Author Contributions

JZ and WS designed the experiments. ShuaiW performed most of experiments and wrote the paper. RZ, YW and JX performed identified the resistance and constructed mapping population. ZS, YaZ, AS, JG, CL, XW, JW, XS, QL, YC, YuZ, and ShuaisW took part in the part of experiments and the paper modification.

## Conflict of Interest

The authors declare that the research was conducted in the absence of any commercial or financial relationships that could be construed as a potential conflict of interest.
