# Supplementary figures and images for: Identification and Fine Mapping of RppM, a Southern Corn Rust Resistance Gene in Maize
Source: Front Plant Sci. 2020 Jul 9;11:1057. doi: 10.3389/fpls.2020.01057 (PMC7363983; doi:10.3389/fpls.2020.01057)

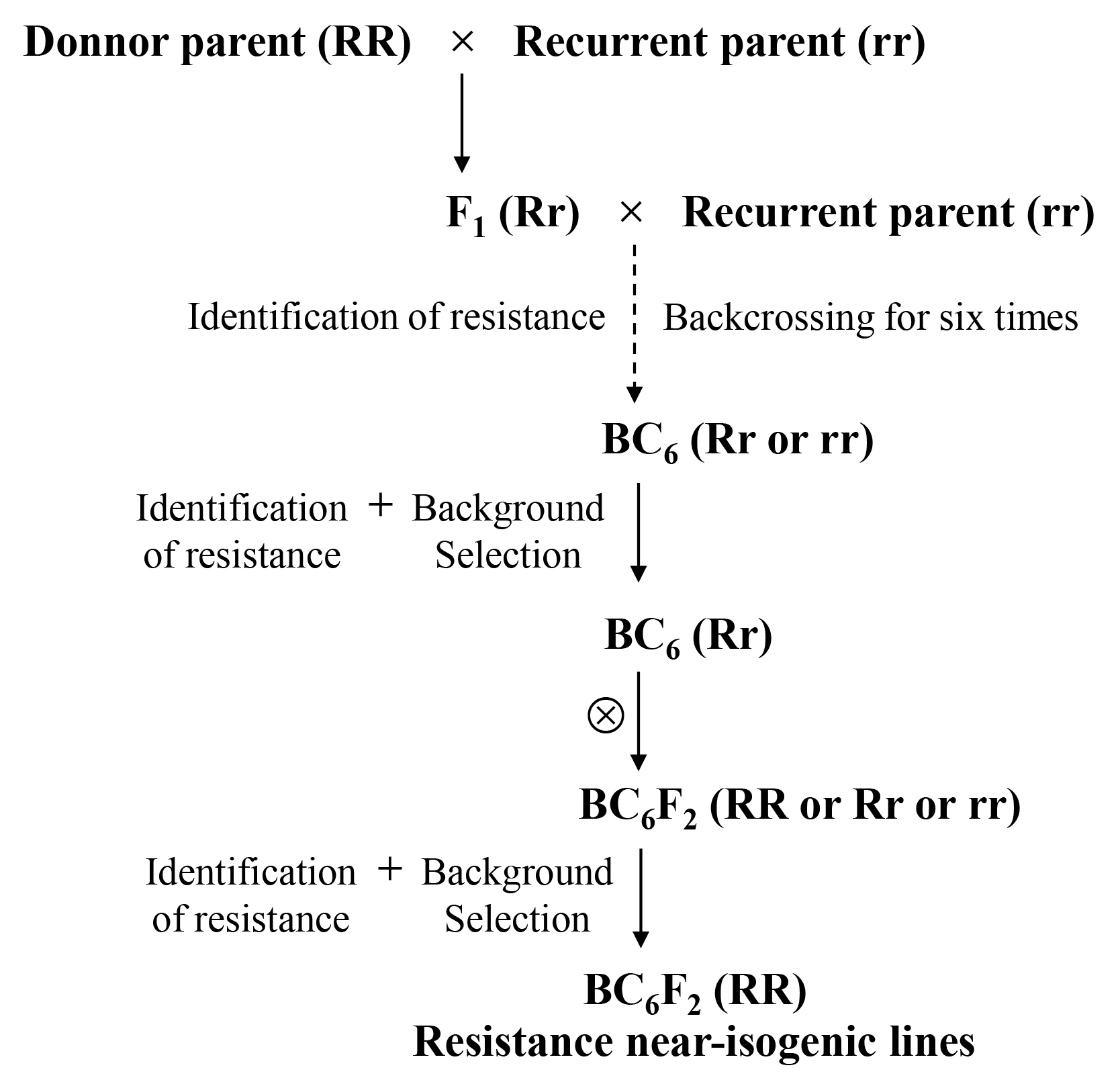

Supplement: Supplementary file 1 [file Image_1.jpeg]

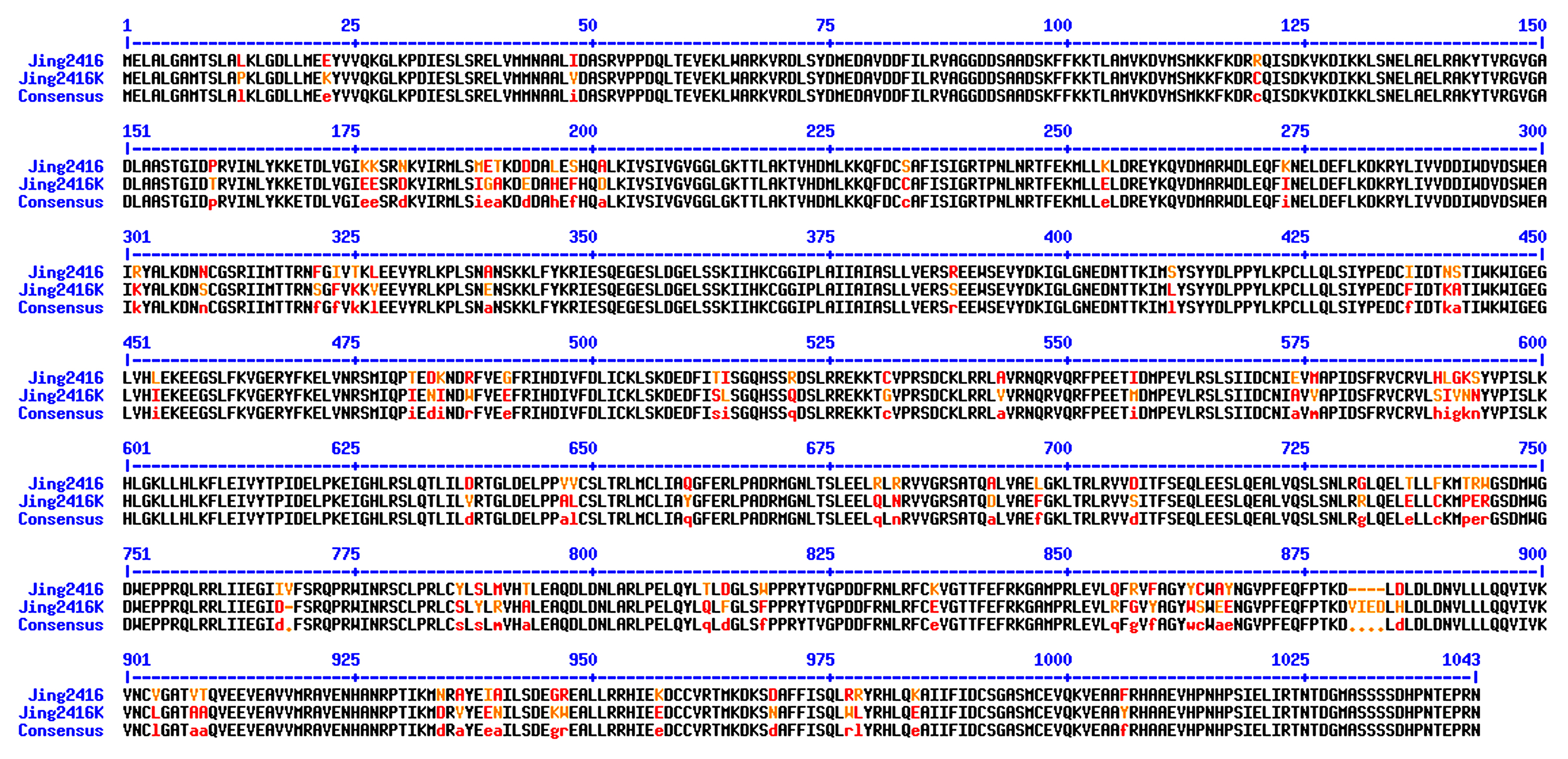

Supplement: Supplementary file 2 [file Image_2.jpeg]

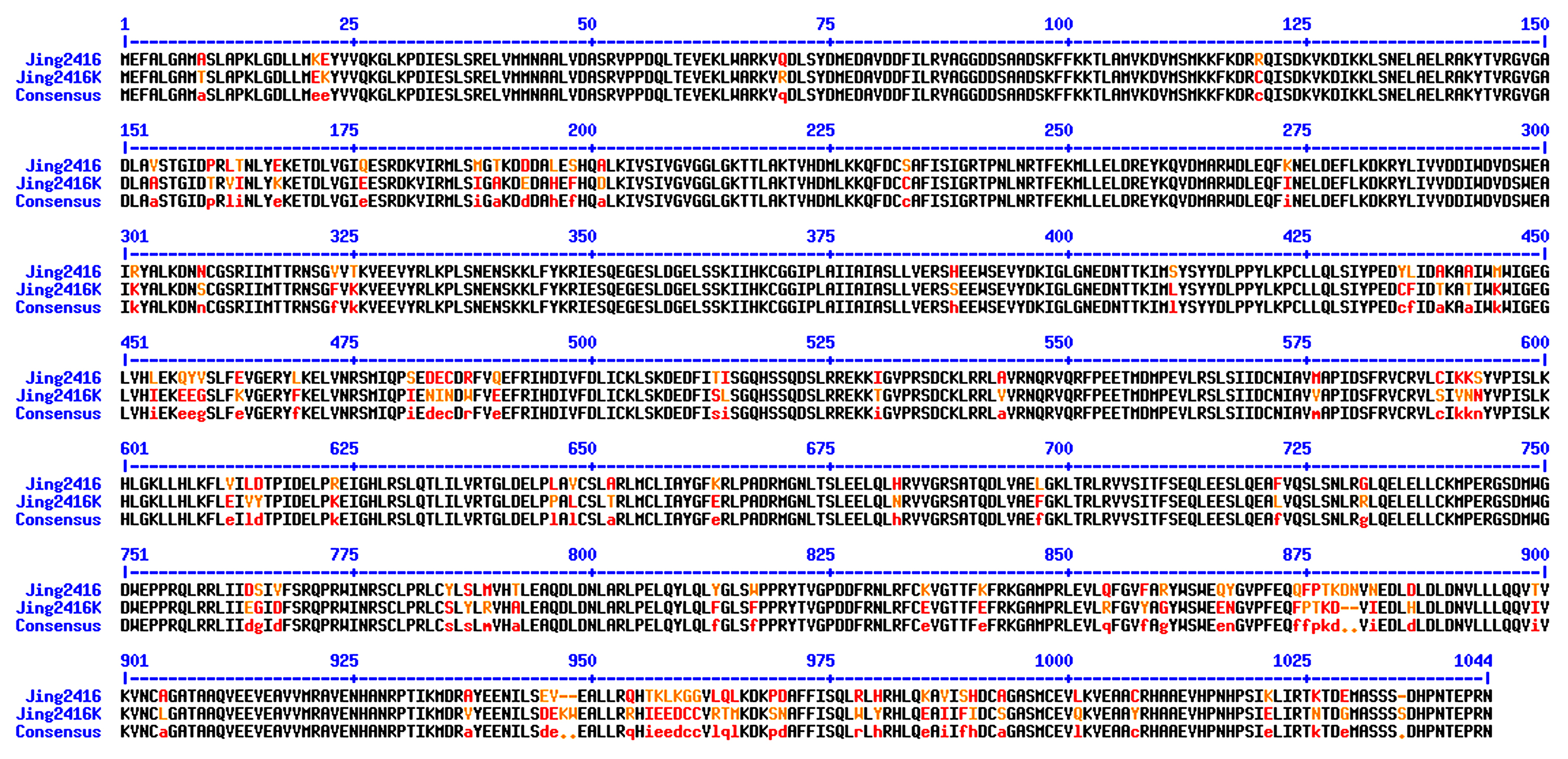

Supplement: Supplementary file 3 [file Image_3.jpeg]
