# Supplementary material for: Identification and Fine Mapping of RppM, a Southern Corn Rust Resistance Gene in Maize
Source: Front Plant Sci. 2020 Jul 9;11:1057. doi: 10.3389/fpls.2020.01057 (PMC7363983; doi:10.3389/fpls.2020.01057)
Supplement: Supplementary file 4 [file Table_1.docx]

Supplementary Tables

**Supplementary Table 1**. Primer sequences of InDel markers used for fine mapping of *RppM*.

| Marker | Physical position (bp) | | Primer sequences（5’ → 3’） | |
| --- | --- | --- | --- | --- |
|  | Start | End | Forward primer | Reverse primer |
| I10-1 | 1052759 | 1052906 | ATTTTGGTTAGTTGGTAT | GCACCCTAAGGTCTTATT |
| I11-1 | 1102871 | 1103014 | GCCCTTGGTTAGTGGTTG | TGCAGCGTTCCAATGTCT |
| I12-2 | 1287777 | 1288011 | CACAAGAAATTGACCCCATCC | TAACAGTATGCTTGCTTA |
| I12-3 | 1247979 | 1248094 | TAGCAGCCATTGATTATGTT | TGCCTGCTCAAGAATCGTAT |
| I13-2 | 1391397 | 1391574 | GTGCTCGATCTGCTGACA | ACCCAGCCTGTGCCTTAT |
| I14-1 | 1461839 | 1462039 | AGCTGGCTTTTCTACGAAGG | ACCCGGATCTGTACATCGAC |
| I14-2 | 1468968 | 1469225 | TTCATAAAGATGCAACTCAT | CGAGTAGGGATTTCTTTCAG |
| I15-1 | 1513514 | 1513756 | GCTATTTATCGGGAACAT | AGCCTGGTTGTAGTATTCA |
| I15-2 | 1562444 | 1562693 | AGTATCTGTCTCCAGGAGGGTC | TGTTCGGAAATGTTGGGTAG |
| I15-5 | 1586583 | 1586728 | GAAGGGTCGGACTCATTT | CCTTGTATAACGGCTTGG |
| I16-4 | 1697170 | 1697418 | CTGGCTGTCGGTACGAACTC | CACGTCACGAATTGACAAGG |
| I17-2 | 1781677 | 1781906 | TGTTCTTGGATATGCTGCTCA | CACAGTTAGGTCTATCAC |
| I17-3 | 1783958 | 1784150 | CCCTCCATGTCGTTTCTTCT | AGCGTGGGAGTGAAGAATGT |
