# Supplementary material for: Identification and Fine Mapping of RppM, a Southern Corn Rust Resistance Gene in Maize
Source: Front Plant Sci. 2020 Jul 9;11:1057. doi: 10.3389/fpls.2020.01057 (PMC7363983; doi:10.3389/fpls.2020.01057)
Supplement: Supplementary file 5 [file Table_2.docx]

Supplementary Tables

**Supplementary Table 2**. Primers used for amplifying CDSs.

| Marker | Primer sequences（5’ → 3’） | |
| --- | --- | --- |
|  | Forward primer | Reverse primer |
| C63 | CTTGAGGAGCAAGAATGACC | AACACGACATAGGGGTAC |
| C64 | GAATCTATCTCCTAACCCTATCAAG | TCTCCTAGCAGGCACATCG |
| C65 | AGGTCGAGGTGGTGAGGT | CATCACTTGGGTCCGTCT |
| C66 | TTGGGAACTCCTATACGGC | GTCGCCAGCAACATTCAG |
| C67 | AGGTGGTGAGGCATGGAGTT | AAACCGAGTCATCAATTCCT |
| C69 | CCCTCAAGCCTCAATACAAACCA | GGAGACGGCAGCTACTCCTTCCTA |
