# Supplementary material for: Identification and Fine Mapping of RppM, a Southern Corn Rust Resistance Gene in Maize
Source: Front Plant Sci. 2020 Jul 9;11:1057. doi: 10.3389/fpls.2020.01057 (PMC7363983; doi:10.3389/fpls.2020.01057)
Supplement: Supplementary file 6 [file Table_3.docx]

Supplementary Tables

**Supplementary Table 3**. Primers used for quantitative real-time PCR.

| Marker | Primer sequences（5’ → 3’） | |
| --- | --- | --- |
|  | Forward primer | Reverse primer |
| RT65 | AATGGTGTGCCGTTCGAG | AGTTGACTTTGACTATTACTTGCT |
| RT67 | CATTACCCTTAGCGGGCAAC | TCTCAACACCTCTGGCATGT |
| Actin | TCCATCATGAAGTGCGACGT | AGTAATCTCCTTGCTCATGC |
