# Supplementary material for: Identification and Fine Mapping of RppM, a Southern Corn Rust Resistance Gene in Maize
Source: Front Plant Sci. 2020 Jul 9;11:1057. doi: 10.3389/fpls.2020.01057 (PMC7363983; doi:10.3389/fpls.2020.01057)
Supplement: Supplementary file 7 [file Table_4.docx]

Supplementary Tables

**Supplementary Table 4**. Overview of sequencing results.

| Sample | Clean Reads | Clean Bases | Q30 (%) | GC (%) | Properly mapped (%) | Average depth |
| --- | --- | --- | --- | --- | --- | --- |
| R01 | 198,650,993 | 59,502,218,156 | 93.33 | 45.78 | 90.49 | 24 |
| R02 | 194,020,377 | 58,107,844,302 | 93.12 | 45.81 | 90.60 | 23 |
| R03 | 261,601,158 | 78,354,498,418 | 93.54 | 45.73 | 89.68 | 32 |
| R04 | 269,312,671 | 80,665,031,218 | 93.27 | 46.06 | 89.65 | 33 |

R01:Jing2416; R02: Jing2416K; R03: the resistant pools; R04: the susceptible pools.
