# Supplementary material for: Identification and Fine Mapping of RppM, a Southern Corn Rust Resistance Gene in Maize
Source: Front Plant Sci. 2020 Jul 9;11:1057. doi: 10.3389/fpls.2020.01057 (PMC7363983; doi:10.3389/fpls.2020.01057)
Supplement: Supplementary file 8 [file Table_5.docx]

Supplementary Tables

**Supplementary Table 5**. Overlapping regions combining SNP-index and InDel-index association algorithms.

| Chromosome | Start | End | Size (Mb) | Gene Number |
| --- | --- | --- | --- | --- |
| 10 | 0 | 3,690,000 | 3.69 | 754 |
| 2 | 0 | 2,950,000 | 2.95 | 532 |
| 2 | 3,020,000 | 3,330,000 | 0.310 | 75 |
| 2 | 3,510,000 | 3,550,000 | 0.040 | 2 |
| 2 | 3,640,000 | 7,030,000 | 3.39 | 723 |
| 9 | 117,570,000 | 119,090,000 | 1.52 | 63 |
| 9 | 119,340,000 | 119,380,000 | 0.040 | 2 |
| 9 | 119,840,000 | 119,900,000 | 0.060 | 7 |
